# The ISCB Student Council Internship Program: Expanding computational biology capacity worldwide

**DOI:** 10.1371/journal.pcbi.1005802

**Published:** 2018-01-18

**Authors:** Jigisha Anupama, Margherita Francescatto, Farzana Rahman, Nazeefa Fatima, Dan DeBlasio, Avinash Kumar Shanmugam, Venkata Satagopam, Alberto Santos, Pandurang Kolekar, Magali Michaut, Emre Guney

**Affiliations:** 1 University College Dublin, Dublin, Ireland; 2 Fondazione Bruno Kessler, Trento, Italy; 3 Genomics and Computational Biology Group, School of Computing and Mathematics, University of South Wales, Treforest, Wales, United Kingdom; 4 Department of Biology, Faculty of Science, Lund University, Lund, Sweden; 5 Computational Biology Department, Carnegie Mellon University, Pittsburgh, Pennsylvania, United States of America; 6 University of Michigan, Ann Arbor, Michigan, United States of America; 7 Luxembourg Centre for Systems Biomedicine, Université du Luxembourg, Luxembourg City, Luxembourg; 8 Novo Nordisk Foundation Center for Protein Research, Copenhagen, Denmark; 9 Strand Life Sciences Pvt Ltd, Bengaluru, India; 10 The Netherlands Cancer Institute, Amsterdam, Netherlands; 11 Joint IRB-BSC-CRG Program in Computational Biology, Institute for Research in Biomedicine, Barcelona, Catalonia, Spain; Genome Quebec, CANADA

## Abstract

Education and training are two essential ingredients for a successful career. On one hand, universities provide students a curriculum for specializing in one’s field of study, and on the other, internships complement coursework and provide invaluable training experience for a fruitful career. Consequently, undergraduates and graduates are encouraged to undertake an internship during the course of their degree. The opportunity to explore one’s research interests in the early stages of their education is important for students because it improves their skill set and gives their career a boost. In the long term, this helps to close the gap between skills and employability among students across the globe and balance the research capacity in the field of computational biology. However, training opportunities are often scarce for computational biology students, particularly for those who reside in less-privileged regions. Aimed at helping students develop research and academic skills in computational biology and alleviating the divide across countries, the Student Council of the International Society for Computational Biology introduced its Internship Program in 2009. The Internship Program is committed to providing access to computational biology training, especially for students from developing regions, and improving competencies in the field. Here, we present how the Internship Program works and the impact of the internship opportunities so far, along with the challenges associated with this program.

## Introduction

Academic training is an important aspect of career building. Students pursuing higher education, in particular, are encouraged from early-career stages to explore opportunities that will assist in both choosing a career path as well as building their skill set. Few things boost a student's career as much as hands-on experience in a field of interest. An internship provides such an experience and is an increasingly important route for students of Science, Technology, Engineering, and Math (STEM) to gain new skills, as well as for employers to find new talent. While internships are commonplace in industry [1], fewer opportunities exist in academia, and yet an internship is especially important for students interested in pursuing an academic career [2]. For undergraduate and graduate students, spending several weeks in an established research lab often strengthens technical competences and soft skills, offers exposure to different perspectives, provides ample networking opportunities [3,4], and plays a crucial role in gaining a competitive advantage as a computational biologist in academia [5].

Benefits of an internship are not limited to the participating student. The labs they work at stand to benefit as well, with the integration of good research practices from other research environments into their own. Bringing ideas together to unite research efforts promotes an inclusive and accessible scientific culture for all. Furthermore, talented and highly motivated students constitute an invaluable potential for group leaders looking for students to undertake projects in their labs. On a global level, knowledge sharing across nations encourages scientific and technological innovation, which in return impacts economic growth.

**Fig 1:**
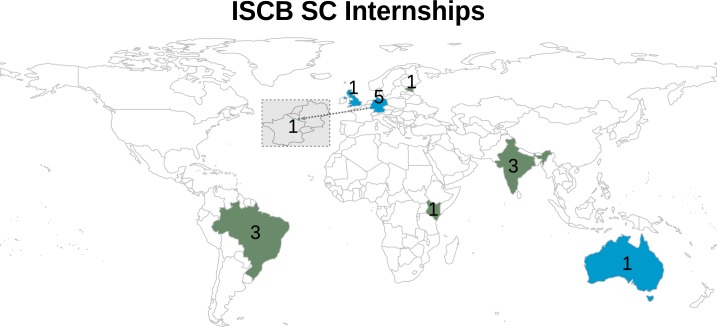
Geographical distribution of the Internship Program participants. The countries where the institutions of the interns and host research labs are located are shown on the map for the participants of the Internship Program as of 2017. The color scale represents the number of internship locations (blue) and home country of the interns (green) that participated in the Internship Program. Luxembourg is highlighted in the inner map, indicated by the arrow. The numbers on the top of the countries correspond to the number of times the country has been the host or country of origin for the intern. The world map was generated using rworldmap R package.

Securing an internship in an established lab can be challenging. Since funding capacity and opportunities are limited, internships in academia are typically scarce. While these challenges are prevalent across the world, the barriers to maximizing career potential are exacerbated for students based in developing nations [6]. This creates a gap in the capabilities, networking opportunities, and competences between young scientists in the developing and those in the developed world. As a result, students from developing nations tend to be less prepared for a career in their field of interest. The need to build and promote the computational biology capacity in developing nations prompted the International Society for Computational Biology Student Council (referred to as the ISCB-SC or simply the SC) [7] to invest effort and resources in creating the Internship Program [8]. With the goal of encouraging equal opportunity for students and future scientists from developing nations, the SC formed the Education and Internships Committee (EIC) to perform this task. The committee organized its first internship in 2009. Since then, the EIC has overseen the coordination of eight internships for students from four developing countries at four research labs located across Europe and Australia (Fig 1).

In this article, we describe the Internship Program’s operation and impact. The article also discusses the associated challenges that need to be addressed and overcome in order to further develop the initiative and continue to build the next generation of computational biology researchers.

## The Internship Program: The EIC’s initiative

**Fig 2:**
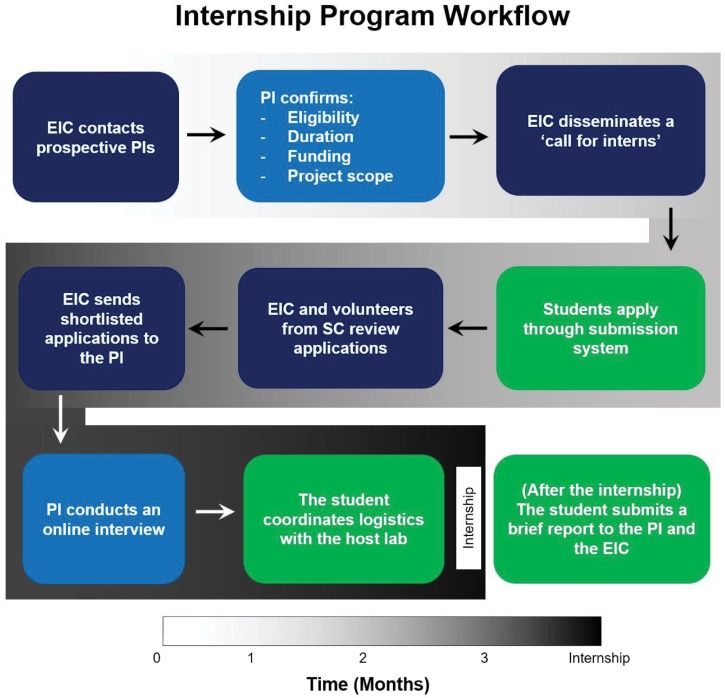
Internship Program: How it works. Steps involved in the internship process. The tasks carried out by the EIC, the PI, and the interns (students) are represented as dark blue, light blue, and green boxes, respectively. First, PIs provide the details of an internship opportunity. Once confirmed, the EIC issues a call for interns and collects applications. Applications are then reviewed, and the shortlisted (up to five) applications are sent to the PI, who makes the final decision. The selected intern makes remaining arrangements with the assistance of the host group. Upon completion of the internship, the student prepares a brief report on her/his research activity and overall experience during the internship. EIC, Education and Internships Committee; PI, principal investigator.

The Internship Program is aimed at improving accessibility to high-quality research for students from developing regions. To this end, the EIC strives to manage the internships in an efficient and transparent manner (Fig 2), providing ease of access for interested principal investigators (PIs). The SC utilizes its vast network and its proximity to the members and leadership of the ISCB to contact PIs interested in the program. Once the PI expresses her/his interest in hosting a student from a developing nation through the Internship Program, he/she is required to provide some information that the EIC can use to prepare an appropriate call for applications. This includes a title and description of the project, the intended duration, prerequisites, and transferable skills that applicants should possess to be eligible to apply for the internship. Additional information on financial support, such as a stipend, travel expenses, and accommodation (if available), is also provided by the host lab because those factors play major roles for students in determining whether they can participate in the program.

The EIC then decides on an operational timeline and opens a call for applications requesting documents such as a cover letter, recommendation letters, certificate of enrollment at an academic institution, and a curriculum vitae. The team mobilizes its principal source of outreach, the Regional Student Groups (RSGs), to circulate the call to as many students as possible [9]. In addition, the Outreach and Volunteers Committee and Web Committee of the SC utilize several mailing lists, social media platforms, and online forums to disseminate the call to computational biology and bioinformatics communities both inside and outside of the ISCB [10–12].

Applications are received through a submission system tailored for the Internship Program by the Web Committee. The applications received before the deadline are reviewed by a review committee—formed by the SC members—based on the criteria provided by the PI and the overall quality of the application. Recommended applicants are forwarded to the PI, who then conducts interviews to select a final candidate. On average, the committee receives over 40 applications for each call, of which up to five are forwarded to the PI.

The overall process of recruitment takes around two months. While deciding the start date of the internship, PIs are advised to take into account the interview procedure and the visa application process, which may take a substantial amount of time for students from developing countries.

## Impact of the Internship Program

Since the beginning of the Internship Program, eight students from developing nations have benefited from research stays in labs across Europe and Australia (Table 1). All the participants have since moved into outstanding positions within computational biology and bioinformatics (Table 1).

**Table 1:** List of internships organized by the EIC since the program’s conception in 2009.

Host Lab	Student’s Home Country	Research Area	Internship Year	Student’s Current Position
Schneider lab, LCSB, Luxembourg	India	Functional annotation of enzymes	2015	Researcher at the Indian Institute of Chemical Biology, India
Bateman lab, EBI, United Kingdom	India	Protein sequence analysis	2014	Research fellow at the University of Leeds, United Kingdom
Ong lab, NICTA, Australia	Brazil	Visualization of genome-wide association study data	2012–2013	Master student at the Federal University of Rio de Janeiro, Brazil
Rost lab, TUM, Germany	Brazil	Protein sequence analysis	2012–2013	PhD student at the Federal University of Rio de Janeiro, Brazil
Rost lab, TUM, Germany	Brazil	Protein sequence analysis	2011	PhD student at the Federal University of Minas Gerais, Brazil
Rost lab, TUM, Germany	Kenya	Protein sequence analysis	2010–2011	Bioinformatician at the International Livestock Research Institute, Kenya
Rost lab, TUM, Germany	India	Protein sequence analysis	2010–2011	Assistant Professor at the King Fahd Medical Research Center, Saudi Arabia
Schneider lab, EMBL-Heidelberg, Germany	Estonia	Text mining for bioinformatics	2010	Research fellow at the Wellcome Trust Sanger Institute, United Kingdom

Abbreviations: EBI, European Bioinformatics Institute; EIC, Education and Internships Committee; EMBL, European Molecular Biology Laboratory; LCSB, Luxembourg Centre for Systems Biomedicine; NICTA, National Information and Communications Technology Australia; TUM, Technical University of Munich.

While the main beneficiary of an internship is the participating student, the home and host labs also tend to benefit from the student’s internship experience. The student acquires valuable research skills and builds new contacts, which could open new doors for their future success. Many of those new skills could be transferred to peers at their home institution upon return from the internship. In a similar fashion, the host lab not only gains new perspectives that come with having a visitor in the lab but also possibly a new collaborator over time. In addition, living abroad even for a short period of time provides insights into new cultures, which is useful when one is collaborating across nations and attending international conferences.

### Impact on the student

The most obvious reward of participation in an internship is the opportunity to hone skills that a smaller or less-established lab cannot provide. Internships help students gain exposure to the current research trends, as well as hands-on access to the latest equipment and facilities that may be unavailable at the home institution of the student. For example, Mohammed Rehan, currently an assistant professor at the King Fahd Medical Research Center in Saudi Arabia, attributes a brand new perspective on his graduate work to the internship in Professor Burkhard Rost's Lab at the Technical University of Munich. Rehan, who spent three months of 2010 in Munich, profited from the discussions at group meetings and guidance from colleagues, which helped shape his project back in India. “The acquired knowledge helped me in how to present my work, asking new questions and designing new projects,” he recollected.

The experience can also expose students to parts of our field they might not have had access to otherwise. Rohit Thakur had the opportunity to work with Dr. Alex Bateman’s group at the European Bioinformatics Institute (EBI) while he was a student at the Vellore institute of Technology in India. The internship led him to discover the breadth of research being carried out in the field of computational biology at a premier research organization. Currently a Marie Sklodowska-Curie research fellow at the University of Leeds in the United Kingdom, Rohit praises his experience as an opportunity that helped establish his research interests and acquire interdisciplinary proficiency in computational biology and programming.

Rohit’s experience also exemplifies another benefit of the program in that it expands career opportunities by making connections that may not have been possible otherwise. Kaur Alasoo, Dr. Reinhard Schneider’s lab intern in 2010 at European Molecular Biology Laboratory Heidelberg, gratefully mentioned his internship experience and emphasized the positive outcome of the program that helped to shape his future opportunities. He added, “The amazing five months are now over, but I am sure that they will keep influencing my life for many years to come…[I] probably will start my PhD in two years…[T]his internship has changed me a lot. It has encouraged me to more eagerly look for opportunities and pursue my research interests.”

Besides the direct benefit of being involved in a cutting-edge research project, being at a well-established research institution carries additional advantages. Academically, these secondary benefits include events such as seminars, symposia, workshops, as well as the diverse everyday interactions that are commonplace at major research institutions. Such events, along with the quality of the research itself, serve as great sources of motivation and inspiration for students, particularly those from developing nations where there may be logistical constraints to the organization of research-oriented events, such as limited funding for science. The secondary benefits are also social; the experience of carrying out research at an institution abroad helps students appreciate the cultural diversity in the country they visit, which contributes to their personal development.

### Impact on peers

In concert with the benefits to the students, the Internship Program creates a unique opportunity for the host lab because the intern will contribute to the diversity of the research group. The internship facilitates interaction between the intern and the graduate students in the host organization and creates an environment for mutual scientific and cultural interchange. The peers have a first-hand and up-to-date perspective on the country of the intern and discuss pressing scientific and sociopolitical issues. In fact, the impact of having an intern from abroad is not only limited to the peers working at the host institution. The peers from other research groups at the host institution also benefit from such intercultural interactions, potentially igniting a reconsideration of stigmas that might be attached to certain countries.

Furthermore, upon completion of the internship, when students return to their home institution, they will undoubtedly share some of the knowledge they have gained abroad. For example, Dedan Githae, another past intern of the Rost lab who participated in the Internship Program from Kenya, refers to his internship as a stimulating and inspiring research environment that provided him transferable knowledge. In his words, participating students “get to learn new and effective skills from leading research groups in developed countries, leading to development and improvement in quality of research back in their own countries. This is not only effective but leads to establishing contacts for the future.”

### Impact on the host PIs

The scientific and cultural exchange between the intern and the host group could also bring novel research questions to attention and initiate collaboration. As a result, it could help PIs to extend their professional and personal network to otherwise less-accessible places to bridge the scientific gap between developed and developing nations.

Dr. Alex Bateman, a host PI in 2014, expressed his appreciation of the program, saying, “I was delighted to take part in the ISCB-SC internship program to help give a hand up to excellent students from around the world.” The participating PIs were also satisfied with the enthusiasm of the students: “The students who joined our group through this Internship Program were very active and eager to learn new things,” were the words of Dr. Reinhard Schneider, who hosted two interns in 2010 and 2015.

Recently, the outreach activities and social impact of a research group have become increasingly important during the evaluation of grant applications. To this end, involvement in the Internship Program provides a valuable opportunity for PIs to reach out to students from developing countries and contribute to the training of the next generation of researchers in the field of computational biology. As a result, this could strengthen the social impact of the research group and center.

From a logistics perspective, the main advantage of this program for a hosting lab is that the EIC facilitates what is otherwise a time-consuming process of finding a motivated and talented student. As outlined above, the EIC acts as an intermediary, advertising the position, evaluating applicants, and providing a list of qualified candidates according to the requirements specified in the offer. Furthermore, the vast reach of the SC network highly increases the probability of finding a suitable candidate for the host lab. Dr. Bateman notes the effective role of the SC in the Internship Program by saying, “The ISCB Student Council made the process very simple and preselected the most able students for me to interview.”

In the long run, the experience of students positively influences computational biology research worldwide and has already had social and scientific impact on their host and home institutions. This includes the transfer of current computational techniques to their research environment, as well as introducing good practices from the host to the home lab, such as task management, resource allocation, and social interactions. Furthermore, participating students pursuing their careers as researchers are likely to participate in initiatives of a collaborative nature in the future, such as the Internship Program [13]. Additionally, many interns have gone on to contribute to various SC activities, such as the creation of RSGs, volunteering at local symposia, and providing similar opportunities to other students.

## Challenges and outlook

The Internship Program has been a rewarding yet challenging initiative to sustain since its inception. The EIC works to liaise between the students from developing nations and research groups from developed nations with the aim of improving equality of opportunity and increasing diversity in the field of computational biology. The Internship Program relies on the support of PIs and the members of the SC for hosting and coordinating the internship opportunities for students from developing countries, placing certain challenges on its sustainability.

### Visibility of the Internship Program

Increasing the visibility of the Internship Program among PIs has been one of the major challenges since the establishment of the program. In an effort to publicize the program, the SC posts information about the Internship Program on its website [14], and the program is advertised extensively through the ISCB and SC networks, as well as on several external platforms such as other professional societies, mailing lists of popular bioinformatics resource portals, and social media. RSGs are also encouraged to promote the Internship Program within their networks and during their annual events.

Despite these efforts, the most effective form of publicity that the program has received so far has been presentations during the annual Intelligent Systems for Molecular Biology (ISMB) [15] meeting and the in-person interactions with PIs by the SC members. Owing to these advertising efforts, the program has thus far received internship position offers from research group leaders who are either ISCB members or whose students work with the SC.

The outreach of the Internship Program could be further increased by organizing workshops, seminars, and career sessions for students across different countries in an effort to reach out to non–ISCB-affiliated potential PIs and interns. During these events, participants would be encouraged to both look out for potential internship positions (if they are eligible) and inform the group leaders they know about the program. To achieve this, the SC aims to leverage the globally spread RSG networks, which typically organize local events and can promote the program among both students and group leaders [9,16]. In addition, the SC will create further publicity at its symposia held in Africa, Europe, the United States, and Latin America via poster presentations. Similar posters will be presented at the main ISCB-affiliated conferences such as the ISMB and European Conference on Computational Biology. Because many of the host PIs in the past have been affiliated with the ISCB, the SC is discussing the possibility of including information on the Internship Program in the membership registration and renewal process to allow members to easily express interest in the program and further publicize it.

### Varying turnout for offered internships

While it is important for the SC to reach a broader community to increase the internship offers, finding the most suitable candidates is also an essential component. Besides securing the best candidate, the SC also strives to balance the participants based on their gender and country of origin. However, the internship offers have seen varying demand from students across different countries. This can be partly attributed to geographical barriers, such as the narrow reach of the RSGs in certain continents like Africa [16], hindering exposure of the internship call to a broad audience in these regions. On the other hand, a lack of foreign language skills, in this case English, can also pose a potential barrier for those who reside in countries where another language (e.g., French) is the most widely spoken language. Accordingly, the SC aims to increase the diversity of the participating countries by expanding its contacts in targeted countries (e.g., finding potential hosts in Canada and France or recruiting students in French-speaking African countries who can translate and promote the internship calls in their country).

On the other hand, the timing of the internship also has a strong effect on the number of students who apply for the position. Internships that span the break between academic years, such as summer months, usually receive more applications than those taking place during the rest of the year because the students cannot accommodate an internship during the academic year. Therefore, the EIC tries to organize internships during the summer to maximize the number of applicants.

### Financial and bureaucratic burden of hosting internships

Among all the challenges that the Internship Program experiences, the financial and bureaucratic burdens of hosting internships are arguably the toughest. The program lacks financial independence and depends entirely on the hosting institution’s funding to support the internship applicants. This may imply allocating budget for stipends for the duration of the internship, travel expenses, and in many cases, expensive visa fees. Consequently, the scarcity of funds could prevent some group leaders interested in participating in the Internship Program. Furthermore, having adequate funds for the aforementioned expenses does not warrant the participation of a PI in the program. Restrictions imposed by funding agencies on how funds can be used or stipulations in some national grants on supporting only students from the host country preclude participation in the program.

Another major challenge that the program faces is associated with tightened visa rules that hamper the movement of people between countries, especially from developing countries. These restrictive rules involve complicated entry requirements, unclear application procedures, and increased transaction costs, which introduce substantial hindrances for both students and PIs and, more importantly, discourage movement and cooperation [17]. Therefore, the success of the Internship Program requires the involvement of public and private institutions—from universities, research centers, and funding agencies to companies, as well as policy makers—to support accessible and agile international exchange in research, especially migration from developing countries as a strategy to make mobility an essential part of human development [17,18].

### Direct challenges on the EIC members

The EIC comprises volunteers of the SC, including both full-time students and early-career researchers, who oversee the coordination of the Internship Program in their spare time. Consequently, during peak times of the Internship Program (e.g., three to four months before summer) when an internship is announced and the selection process takes place, the program-related tasks tend to be demanding for the committee members. This creates a workload imbalance for the volunteers, requiring more time commitment during certain months and less-intensive dedication for the rest of the year. To tackle this issue, the EIC usually establishes ad hoc evaluators for each internship position, by which volunteers from other committees or RSGs participate in assessing applications—determining the eligibility of the applicant, interpreting official documents issued by the home institution (e.g., official certificate of registration in a university, reference letters), performing quality checks, and performing skill evaluation. The EIC also tries to optimize its communication workload from applicants by providing a clear definition of the requirements and answers to potential (frequently asked) questions students might have.

In an attempt to provide more benefits to the community, as well as to motivate volunteers, the EIC is working on introducing new initiatives, such as building a resource portal for computational biology education. The portal will be an open source online knowledge base dedicated to bioinformatics and computational biology researchers across the globe. The resource is planned to contain various tutorials and exercises ranging from basic to advanced bioinformatics topics and algorithms and the latest journal articles and books, and it will provide an interface to submit personalized materials (e.g., articles, tutorials, videos) to assist researchers. The goal of this portal is not to replicate the efforts of ISCB Education Committee [19] and the ISCB Computational Biology Education Community of Special Interest (CoBE COSI) [20] but to provide specialized resources tailored to students. Our hope is that, in the future, we will be able to work together with these organizations to benefit all members of our community. We believe this will empower the role of the committee in computational biology education and training and help to incorporate more volunteers who can assist during the peak times of the Internship Program. Currently, there are four core members who are active all year round and around 10 student volunteers, who form an ad hoc committee to evaluate applications when necessary. The ISCB supports the concept and the activities of the EIC, providing feedback but also leeway on how the internships are managed and reviewed.

### Future perspectives of the Internship Program

The Internship Program plays a key role in bridging the gap in computational biology research between developing and developed countries. The program relies on the host labs to sponsor students from developing nations, and the SC strives to minimize the effort and time commitment from a PI for recruiting an intern by streamlining the entire pre-internship process, from advertising the position and collecting applications to screening the applications. The breadth of the SC and affiliated RSG network ensures that the calls for applications reach the countries where these RSGs are located [21]. The extensive student network also makes it easier to find volunteers who build and maintain application submission systems, as well as get reviewers for incoming applications. Encouraged by not only the support from numerous SC volunteers but also the positive response from both students and PI alumni alike, the SC is motivated to sustain the initiative and make a lasting positive impact on the computational biology society.

To ensure the continuity and success of the Internship Program, the SC aspires to engage more PIs and students to contribute to the program. Ideally, the EIC would like to increase the number of positions per year steadily, but this depends on resources, such as the availability of funded positions, the number of students interested in applying for them, and volunteers dedicated to their handling. Currently, the SC leverages various ISCB conferences [22], SC’s symposia, and RSG activities to expand the breadth of the publicity and visibility of the program and available internship offers.

The support of an influential professional body such as the ISCB, which plays a major role in advancing the field of computational biology, becomes crucial in taking the Internship Program initiative forward. The ISCB has helped establish the SC, and its leadership continues to provide guidance, support, and financial aid, as well as promote various SC activities. Gauging the potential of the Internship Program, the ISCB has recently announced that it will provide funding to support travel costs for internship positions offered by ISCB-affiliated PIs [23] through the Anna Tramontano fellowship fund. Established to honor the memory of Anna Tramontano, a renowned computational biologist and long-time ISCB member, this fellowship fund accepts donations. The SC expects that this dedicated fund for the Internship Program will attract PIs who have the academic resources and are interested in participating in the program but lack the financial capital to host interns. The SC welcomes other potential collaborations and input from research institutions, professional societies, and corporate organizations in continuing the program and making it more sustainable.

The SC also seeks to increase the number and expand the diversity of students benefiting from the program by trying to accommodate the needs of students across different countries. Accordingly, the SC tries to offer internship opportunities during the largest interval in the academic calendar, facilitating the participation of students who are enrolled in courses during the fall and spring semesters. To expand the reach of the program to countries where French rather than English is the second language, the SC looks forward to reaching out to PIs from French-speaking developed countries as potential hosts.

The Internship Program has a well-defined organizational structure that has seen success over the past eight years, placing eight students from developing nations as interns in four research labs across Europe and Australia. The internship positions kindly offered by a number of PIs have created a monumental impact on the careers of the participating students, prompting the SC to ensure the continuity of the Internship Program. However, various factors put its sustainability in jeopardy, including scarce funding, difficulties in advertising, limited participation, and bureaucracies associated with international travel for the interns. Some of these roadblocks are more easily addressed than others. To address the lack of participation, more host labs are needed to step forward and contribute to initiatives like the Internship Program, and on the other hand, students must realize the importance of exposure beyond classroom lectures and actively look for opportunities to broaden their skills. The SC is dedicated to launching the careers of more students from developing countries, and together with the support and encouragement from sponsors and international research organizations, the initiative holds great potential to shape the future of the field of computational biology.
